# Correction to “Neurofilament light chain levels in cerebrospinal fluid as a sensitive biomarker for cerebral adrenoleukodystrophy”

**DOI:** 10.1002/acn3.51870

**Published:** 2023-08-17

In the originally published version of the article, the patient labeling in the Results section on page 6 was incorrect and this has been corrected as follows.

From:

Cognitive function deteriorated in two patients (patients 20 and 21), while it was preserved in the other two patients (patients 17 and 18).

To:

Cognitive function deteriorated in two patients (patients 20 and 21), while it was preserved in the other two patients (patients 18 and 19).

In the originally published version of the article, there were labeling errors in the Figure 5B.

Figure 5B has been replaced with the corrected version shown here. 

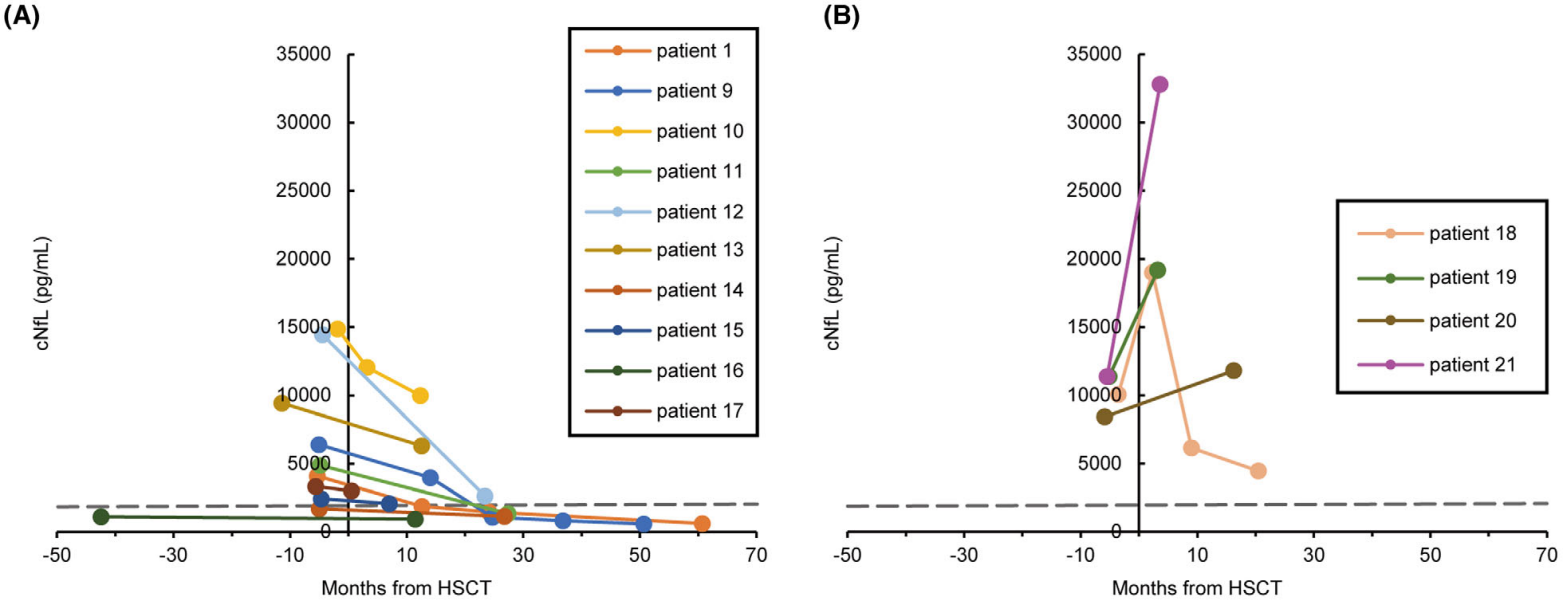

We apologize for these errors.

Kakumoto T, Matsukawa T, Ishiura H, Mori H, Tsuji S, Toda T. Neurofilament light chain levels in cerebrospinal fluid as a sensitive biomarker for cerebral adrenoleukodystrophy. Ann Clin Transl Neurol. 2023. https://doi.org/10.1002/acn3.51818

